# Physician-facilitated designation of proxy decision maker

**DOI:** 10.1186/s13584-016-0084-5

**Published:** 2016-06-28

**Authors:** Amit Arora, Laura Cummings, Peter Crome

**Affiliations:** University Hospital of North Midlands, Stoke on Trent, UK; Keele University, Keele, Stoke on Trent, Staffordshire UK; University College, London, UK

## Abstract

With vast improvements in healthcare in recent decades, people are living longer but often with higher rates of morbidity and chronic illnesses. This has resulted in a higher proportion of the population who may benefit from early end-of-life ‘conversation and planning’, but also gives healthcare professionals more time during which these discussions are relevant, as people live longer with their chronic diseases. A survey conducted by Lifshitz et al (Isr J Health Policy Res 5:6, 2016) sought to assess physician awareness and willingness to discuss designating a proxy decision-maker with patients, in order to aid end-of-life care in the event that the patient is rendered unable to make or communicate these decisions later in life.

Their article suggests that proxy decision-maker designation is only one aspect of end-of-life care; a challenging area littered with ethical and moral dilemmas. Without early, open and frank discussions with patients regarding their wishes at the end of life, proxy decision-makers may be in no better position than physicians or a court appointed proxy to make decisions in the patients’ best interests/benefits. This commentary also touches upon the use of health and care passports being developed or in early phases in the United Kingdom, and whether these may be utilised in the field of palliative care in Israel.

## Background

Palliative care is a relatively new medical specialty, experiencing a period of rapid growth and development following the opening of St. Christopher’s Hospice in London, the first of its kind, in 1967 by Cicely Saunders (http://www.stchristophers.org.uk/about/history/pioneeringdays). The principles of palliative care centre on holistic care of the person who is usually in the final phase of life, including addressing psychological and spiritual needs, as well as enabling the patient to live as actively as they wish until death [1]. It is important to note that, where formal palliative care services exist it is not confined to patients who are deemed end-of-life, although that is primary focus of this commentary. In Israel, palliative care is yet to establish itself as a board-certified specialty.

It is not just the clinical management of patients approaching towards end-of-life that is challenging. Decision making regarding place of care, hospitalization, artificial feeding, intravenous fluid replacement, intubation, preferred place of dying, and even cardiopulmonary resuscitation can be extremely sensitive issues. One should endeavour to involve patients in making all of these decisions, wherever possible. But what should be done when patients are unable to communicate these wishes? Consideration of these issues often starts too late on in the patient journey, and quite often the patient has already reached a stage where they can not communicate their wishes.

### Key findings and recommendations from Lifshitz and colleagues

One option to aid healthcare professions in addressing end-of-life issues is to facilitate the designation of proxy-decision makers (PDM), often a relative or close friend of the patient. The survey conducted by Gideon Lifshitz [2] and colleagues published in this issue sought to assess how willing primary care physicians were to explore this option with their patients, at what point they thought it appropriate, and the perceived issues in doing so. Some of their results are quite interesting.

When asked at what point in a patient’s journey the primary care physicians felt it appropriate to discuss the appointment of a PDM, 91 % felt this was relevant when patients were suffering from severe chronic conditions, such as cancer or heart failure. However, only 24 % felt this was an appropriate discussion to have with ‘**all’** elderly patients. This seems a somewhat surprising result. The World Health Report, 2003 [3] estimated that dementia contributed 11 · 2 % of years lived with disability in people aged 60 years and older; more than stroke (9 · 5 %), musculoskeletal disorders (8 · 9 %), cardiovascular disease (5 · 0 %), and all forms of cancer (2 · 4 %). These figures, combined with the knowledge that the disease course of dementia itself will eventually render a person unable to make decisions with capacity, suggest that dementia may be one of the most common scenarios in which a person may benefit from having designated a PDM. In the cases of those with living with cancer or heart failure, the point of diagnosis can act as a prompt to discuss the appointment of a PDM. In those living with dementia, the point at which diagnosis is made may not give enough time to discuss the important end-of-life issues, due to a lack of capacity. Having this discussion with all elderly patients may be a way to address this issue, prior to or early on at the time of diagnosis of chronic cognitive impairment.

### Addressing the challenge of shifts in patient preferences

One of the main perceived impediments to discussing the appointment of a PDM was that primary care physicians felt that patients may change their minds in later life. This could be true regardless of any planning undertaken to prepare for end of life issues. Advance statements, or a person acting as a PDM, would both need regular review and updating regarding patient preferences. In Israel, there is a distinction made between a court appointed proxy, known as a legal guardian, and patient designated proxies, usually a friend or relative.

However, when a proxy is appointed, this does not negate the need to discuss these issues with patients early on. Although the designated person may be a friend or relative, this does not suggest they would necessarily know what the patient would want in terms of feeding, care provision, or place of death. Without facilitation or encouragement of those discussions, proxy-decision makers may be left in a stressful and uncomfortable position, where they feel the responsibility of making important end of life decisions for someone, despite having never been able to talk with the patient about these specific topics. We propose why should not these early discussions about ‘end of life plans’ be called ‘rest of life plans’ instead? Would it help in making these discussions somewhat easier and more acceptable in society?

### Economic considerations

Controversially, one of the perceived benefits of early discussion of a PDM that emerged in this survey was around the cost of service provision at the end of life. Lifshitz et al. commented that currently, if no advanced planning has taken place, the medical profession is more likely to provide all treatments available, rather than palliation early on, despite a sense of futility in certain cases. This may well be the case, but quite often it is the physician who is able to identify the patient who is entering the final phases of life, and when physician led discussions do take place regarding withdrawal of treatment and palliation, this may meet resistance from the family or friends of the dying patient,. There is often a sense of turmoil, uncertainty, and occasionally guilt emanating from the family and friends of such patients. Family and friends would generally want to do what they think the patient would have wanted, but when unable to ask their loved one about their preferences, they feel better by trying to do more. The designation of a PDM alone may not reduce the unwanted, costly, and sometimes undignified, medical interventions. Discussions with the patients early on, however, may help in doing so.

Due to their sensitive nature, such decisions will often require more than one or two meetings and there will still remain a need to review these periodically. In our experience, these meetings are likely to be much longer than standard outpatient clinic appointments and are probably best carried out in an inter-disciplinary environment incorporating family members, carers, nurses, therapists and with agreement from patients’ usual family physician.

Physician reimbursement schemes may also need to reflect what best practice is. In the UK general practice, there is an incentive for the general practitioners to make a summary advance care plan [4] for the top two percent of patients at risk of hospital admission These patients are generally older people with multi-morbidity. This patient-held record summarizes the patients’ medical details and requires the physician to hold regular discussions with the patient regarding advanced care planning. This includes their preferences regarding place and type of care or interventions they may want or not want when their health deteriorates. There is an expectation that these will be updated periodically.

In a commentary by Kinzbrunner and Kinzbrunner, the emerging field of spiritual care in Israel recognises the importance of a wide range of healthcare professionals, to include nurses, social workers and rabbis, in providing spiritual care to those with oncological or palliative diagnoses [5]. It may be appropriate to involve this same group of care providers should a similar advance care planning tool be adopted in Israel. Chaplains of all faiths are available to patients in UK hospitals; however they tend not to be involved in healthcare or end of life decisions, with an extremely limited role in the multidisciplinary team at present.

In the UK, there is an on-going debate as to which care provider is best placed to have these discussions – primary or secondary care. Essentially, both levels of care should play important roles in advanced planning, and all contact with healthcare providers should be seen as an opportunity to have such discussions when appropriate and able.

### Recent UK developments

In the UK, people are able to appoint a Lasting Power of Attorney for health and welfare, providing legal documentation of designated proxy decision makers. Once appointed, this directive remains in place for the duration of the patients’ lifetime. It is now recognised that more formal documentation of patients and relatives wishes would be an invaluable addition to this.

There are multiple types of healthcare ‘passports’ at varying stages of development, and in certain specialties, such as obstetrics and paediatrics, these documents are already in widespread use. More recently, we have developed a comprehensive document; a ‘©Rest of life care passport ‘or ‘©Frailty Passport’ [Rest of Life Care Passport, Amit Arora, Unpublished data, forthcoming] to summarize these discussions and ease the patient journey through various providers and transitions of care. This aims to reduce the need for repeated and often ‘piece-meal’ discussions by various professionals, helps with seamless journey through transitions of care between primary, secondary, social care interfaces and between different geographical locations. It also has been noted to be useful in reducing length of stay in those patients who get admitted to hospitals repeatedly and often get ‘stranded’. It includes a summary of medical conditions, a list of the key care providers, an emergency care plan, patients’ wishes, and agreements around medical and social care plans, next of kin details and details of PDMs as nominated by the patients. It also aims to cover certain important end of life issues (Box 1) that patients may feel strongly about, giving them the opportunity to make their wishes known in advance. There is a need to share these and keep them reviewed on a regular basis. It is hoped that for such conversations to be effective, they should start to take place with in last couple of years of life or in very early stages of dementia onset.

A commentary by Cassileth in 2012 [6] describes the progress achieved by the Middle East Cancer Consortium set up in Israel to improve end of life care. One route they have taken has been to hold annual meetings to help educate and promote palliative care throughout the Middle East. There will be differing ideas across countries regarding the importance of all aspects of end of life care, including emotional and spiritual wellbeing and religious beliefs may also play an important part in this. An ‘A Rest of Life passport’ may be appropriate to adopt to provide a framework of areas to consider for a patient who is approaching the end stages of life. This may help to provide some level of standardisation and yet remain flexible enough to be transferable to most population groups.

Box 1 Some suggested areas to be covered in a ‘Frailty Passport’®

**Table:** 

Nasogastric tube feeding or PEG feeding to prolong life Intravenous or subcutaneous fluids to prolong life Admission to Intensive care/mechanical ventilation/dialysis/surgery etc. Advance statements about wishes and preferences Religious preferences Preferred place of death Proxy decision maker details and next of kin details	

## Conclusions

In this relatively new and growing specialty, there are sensitive and difficult issues to broach, both with patients and their loved ones. Early, open and honest discussions to encourage consideration of these issues are a vital first step in planning for the palliative phase of life. Communication skills development and education early in medical training will help. However, this will also require societal education to reduce stigma and increase awareness around difficult conversations around end of life issues.

## Abbreviations

PDM, proxy decision maker; NG, nasogastric; PEG, percutaneous endoscopic gastrostomy
